# Effect of trace elements and nutrients on 21 autoimmune diseases: a Mendelian randomization study

**DOI:** 10.3389/fimmu.2024.1462815

**Published:** 2025-01-20

**Authors:** Ming-Jie Jia, Hua-Fang Yin, Ying-Chao Liang, Feng Jiang, Hui-Lin Li

**Affiliations:** ^1^ The Fourth Clinical Medical College of Guangzhou University of Chinese Medicine, Shenzhen, Guangdong, China; ^2^ Shenzhen Traditional Chinese Medicine Hospital Affiliated to Nanjing University of Chinese Medicine, Shenzhen, Guangdong, China; ^3^ Ruikang Hospital Affiliated to Guangxi University of Chinese Medicine, Nanning, Guangxi Zhuang Autonomous Region, China; ^4^ Department of Endocrinology, Shenzhen Traditional Chinese Medicine Hospital, Shenzhen, Guangdong, China

**Keywords:** Mendelian randomization, autoimmune diseases, trace elements, systemic lupus erythematosus, autoimmune

## Abstract

**Background:**

Numerous clinical studies have observed a close relationship between serum trace elements, nutrients, and autoimmune diseases. However, whether there is a genetic causal effect between serum trace elements, nutrients, and autoimmune diseases remains unclear.

**Objective:**

This study aims to investigate the causal effects of serum trace elements and nutrients on 21 autoimmune diseases using Mendelian randomization (MR).

**Methods:**

Single nucleotide polymorphisms for the exposure factors (serum trace elements and vitamins) were obtained from the published UK Biobank database and genome-wide association study (GWAS) public databases. Outcome GWAS data were derived from the FinnGen database. MR was employed to explore the causal relationships between 9 trace elements and 6 vitamins and autoimmune diseases. Causal inference was performed using inverse variance weighted methods, MR Egger, and weighted median methods. Subsequently, heterogeneity tests, horizontal pleiotropy tests, MR-PRESSO tests, and leave-one-out analyses were conducted for sensitivity analysis to evaluate the robustness of the study results. Finally, trace elements and vitamins that were statistically significant in the IVW method and had consistent effect sizes and odds ratios across five methods were selected as exposure factors with a causal relationship to diabetes and its complications. Additionally, multivariable Mendelian randomization was employed to assess the combined effects of multiple exposure factors on autoimmune diseases.

**Results:**

MR analysis indicated that elevated levels of the trace element copper were associated with an increased risk of systemic lupus erythematosus (SLE) and a decreased risk of ulcerative colitis. Carotene was found to have a negative causal relationship with adult-onset Still**’**s disease (AOSD). Elevated levels of copper and selenium were associated with an increased risk of autoimmune hyperthyroidism. Calcium levels showed a negative causal relationship with the risk of polyarteritis nodosa. MVMR results demonstrated that selenium could independently affect the risk of autoimmune hyperthyroidism, separate from copper.

**Conclusion:**

The findings from both univariable and multivariable Mendelian randomization studies support a causal relationship between trace elements, nutrients, and autoimmune diseases. These results have significant clinical implications for developing targeted prevention and treatment strategies for autoimmune diseases.

## 1 Introduction

Autoimmune diseases constitute a group of disorders precipitated by aberrant immune system function, and their incidence is on a continual rise. These conditions inflict damage on multiple organs and systems, encompassing neurological disorders such as multiple sclerosis and myasthenia gravis, endocrine diseases including Hashimoto’s thyroiditis and type 1 diabetes, as well as connective tissue disorders like rheumatoid arthritis and systemic lupus erythematosus (1–3). Among adults aged 20 to 79 afflicted by autoimmune diseases, multi-system damage represents the predominant cause of mortality (4). Systemic lupus erythematosus (SLE) is one of the most prevalent autoimmune diseases, characterized by the immune system erroneously targeting the body’s normal tissues. SLE can affect multiple organs and systems, including the skin, joints, kidneys, heart, lungs, and brain (5). Rheumatoid arthritis (RA) is a chronic autoimmune disorder marked by persistent synovial inflammation, which leads to destructive tissue outcomes. RA frequently results in joint damage and functional impairment, impacting approximately 0.2-1% of the global population (6). RA is a principal cause of disability and workforce loss worldwide and a significant risk factor for cardiovascular disease, substantially elevating the risk of cardiovascular events, infections, and mortality (7). Type 1 diabetes is an autoimmune disease wherein the immune system attacks pancreatic islet cells, resulting in insufficient or entirely absent insulin secretion. As of 2021, approximately 8.4 million individuals globally were affected by type 1 diabetes, with projections indicating this number will rise to between 13.5 million and 17.4 million by 2040 (8). Type 1 diabetes can lead to a myriad of chronic complications, including cardiovascular disease, neuropathy, retinopathy, and nephropathy, severely impacting patients’ health and quality of life (9). The repercussions of autoimmune diseases extend beyond significant health impacts on patients to impose substantial socioeconomic burdens on a global scale (10). Hence, it is imperative to explore strategies for early prevention and intervention.

The impact of trace elements and vitamins, collectively known as micronutrients, on autoimmune diseases has garnered considerable attention. Research indicates that deficiencies or excessive intake of certain micronutrients are closely associated with the onset and progression of autoimmune diseases. For instance, a daily intake of 2000 International Units (IU) of vitamin D can reduce the incidence of autoimmune diseases by 22% (11). Higher vitamin E intake is correlated with lower prevalence rates of subclinical hypothyroidism and autoimmune thyroiditis in men (12). Systematic reviews and meta-analyses have found that serum and plasma zinc concentrations are significantly lower in patients with autoimmune diseases compared to control groups (13). Regular vitamin E supplementation can help individuals with RA alleviate joint discomfort, swelling, and stiffness, thereby enhancing their overall quality of life (14). However, observational studies on the correlation between micronutrient levels and disease severity yield inconsistent results. For example, some observational studies suggest an inverse relationship between vitamin D levels and SLE activity, while others do not observe this association (15). Regarding the relationship between vitamin D levels and SLE disease activity, no consensus has been reached. Some studies indicate an inverse correlation between vitamin D levels and SLE activity, whereas others do not find such a relationship. Several studies also reveal that the majority of MS patients exhibit vitamin D deficiency. Specifically, low serum 25(OH)D levels are associated with increased disability and relapse rates in MS patients; however, the biologically active form of vitamin D, serum 1,25(OH)2D, does not show a direct association with disability and relapse rates. Reports by Munger and Kragt et al. highlight a strong inverse relationship between serum 25(OH)D levels and MS risk; nevertheless, this correlation is observed only in Caucasians and not in African Americans or Hispanics (16, 17). Given the inherent design characteristics of observational studies, they are susceptible to various confounding factors (such as confounding variables, time sequences, socioeconomic factors, etc.), which may compromise the accuracy and reliability of the findings. Copper, selenium and carotenoids play important roles in the immune system as key trace elements. Copper enhances the immune response by participating in the antioxidant response and promoting the growth and function of immune cells. In particular, copper is critical to the immune system during the activation of macrophages and T cells. Selenium acts primarily through selenoproteins, which regulate the balance of the immune response and participate in the antioxidant response, helping to reduce the inflammatory response and enhance immune cell function. Selenium deficiency weakens the immune system’s defenses. Carotenoids, as potent antioxidants, promote immune cell function by reducing oxidative damage, modulate cytokine levels in the immune system, and inhibit inflammatory responses, thereby preventing the development of chronic inflammation. These micronutrients work together to maintain the health and homeostasis of the immune system through the multiple mechanisms of antioxidation, modulation of immune response and reduction of inflammation. Therefore, more robust research methodologies are required to elucidate the specific causal relationships between micronutrients and autoimmune diseases.

With the increasing prevalence of large-scale genome-wide association studies (GWAS), Mendelian randomization (MR) has emerged as a potent tool for causal inference across different phenotypes (18). MR is a methodology that integrates epidemiology and genetics by leveraging naturally occurring genetic variations to simulate randomized experiments, thereby enabling more precise estimation of the impact of specific factors on outcomes (19, 20). In MR, single nucleotide polymorphisms (SNPs) associated with the exposure event serve as instrumental variables. Because these instrumental variables are independent of other confounding factors, MR can assess the causal relationships between previously observed exposures and outcomes while effectively circumventing the confounding bias inherent in traditional epidemiological studies (21). Multivariable Mendelian randomization (MVMR) builds on the foundation of MR by considering the causal relationships between multiple exposure factors and disease. Through this approach, MVMR studies can evaluate the joint effects of multiple exposures on disease risk, as well as the interactions among these exposure factors (22, 23). In this context, our study employs both two-sample MR and MVMR methodologies to assess the impact of nine trace elements and six nutrients on the occurrence of autoimmune diseases. The anticipated findings are expected to provide novel scientific evidence and strategies for the clinical application of micronutrients in the prevention and treatment of autoimmune diseases.

## 2 Materials and methods

### 2.1 Data sources

The genome-wide association study (GWAS) data for trace elements and nutrients used in this study were obtained from the IEU OpenGWAS database (https://gwas.mrcieu.ac.uk/). Detailed GWAS information is provided in **Table 1**. The GWAS information for the 21 autoimmune diseases was retrieved from the Finnish database (https://www.finngen.fi/en/access_results). The diseases under investigation include: type 1 diabetes, systemic lupus erythematosus, IgA nephropathy, adult-onset Still’s disease, multiple sclerosis, polymyositis, Guillain-Barré syndrome, autoimmune thyroiditis, autoimmune hyperthyroidism, autoimmune hypothyroidism, small intestine Crohn’s disease, large intestine Crohn’s disease, ulcerative colitis, interstitial lung disease associated with systemic autoimmune diseases, ankylosing spondylitis, psoriasis vulgaris, rheumatoid arthritis, myasthenia gravis, Ménière’s disease, polyarteritis nodosa, and erythema nodosum. Detailed disease data information is presented in **Table 2**.

**Table 1:** GWAS information for trace elements.

Trace Element	GWAS ID	Race	Sample Size	Number of SNPs	Year
Copper	ieu-a-1073	European	2603	2543646	2013
Calcium	ukb-b-8951	European	64979	9851867	2018
Selenium	ieu-a-1077	European	2603	2543617	2013
Zinc	ieu-a-1079	European	2603	2543610	2013
Carotene	ukb-b-16202	European	64979	9851867	2018
Folate	ukb-b-11349	European	64979	9851867	2018
Iron	ukb-b-20447	European	64979	9851867	2018
Magnesium	ukb-b-7372	European	64979	9851867	2018
Potassium	ukb-b-17881	European	64979	9851867	2018
Vitamin A	ukb-b-9596	European	8863	460351	2018
Vitamin B12	ukb-b-19524	European	64979	9851867	2018
Vitamin B6	ukb-b-7864	European	64979	9851867	2018
Vitamin C	ukb-b-19390	European	64979	9851867	2018
Vitamin D	ukb-b-18593	European	64979	9851867	2018
Vitamin E	ukb-b-6888	European	64979	9851867	2018

**Table 2:** Corresponding GWAS information for diseases.

Disease Name	phenotype	Race	sample size	control subjects
Type1 diabetes, definitions combined	T1D	European	4196	308252
Lupus erythematosus	L12_LUPUS	European	652	353088
IgA nephropathy (IgA glomerulonephritis)	N14_IGA_NEPHROPATHY	European	592	376685
Adult-onset Still disease	STILL_ADULT	European	113	341455
MS-disease/Multiple Sclerosis	G6_MS	European	2182	373987
Polymyositis	M13_POLYMYO	European	225	365533
Guillain-Barre syndrome	G6_GUILBAR	European	415	370790
Autoimmune thyroiditis	E4_THYROIDITAUTOIM	European	489	320703
Autoimmune hyperthyroidism	AUTOIMMUNE_HYPERTHYROIDISM	European	1828	279855
Hypothyroidism, autoimmune,	E4_HYTHY_AI_DX	European	92	376788
Crohn’s disease of small intestine	CHRONSMALL	European	2004	359927
Crohn’s disease of large intestine	CHRONLARGE	European	1581	359927
Ulcerative colitis (strict definition, require KELA, min 2 HDR)	K11_UC_STRICT2	European	5034	371530
ILD related to systemic autoimmune disease	ILD_SYST_AUTO	European	104	373064
Ankylosing spondylitis	M13_ANKYLOSPON	European	2860	270964
Psoriasis vulgaris	L12_PSORI_VULG	European	5759	364071
Rheumatoid arthritis	M13_RHEUMA	European	12555	240862
Myasthenia gravis	G6_MYASTHENIA	European	426	373848
Meniere’s disease	H8_MENIERE	European	2746	359094
Polyarteritis nodosa	M13_POLNODOSA	European	185	365533
Erythema nodosum	L12_ERYTHEMANODOSUM	European	814	364583

### 2.2 Study design

In choosing the P-value threshold for SNPs, we used 5 × 10-^6^ rather than the more stringent 5 × 10-^8^. The reason for choosing 5 × 10-^6^ is that a more stringent threshold may result in screening too few SNPs, thereby reducing the statistical power of the study, especially in multiple autoimmune disease analyses. In addition, 5 × 10-^6^ as a threshold has been widely used in many similar Mendelian randomization studies in accordance with the STROBE-MR study specifications, and there is already literature supporting this criterion. By choosing this threshold, we were able to balance the number and statistical significance of the screened SNPs to ensure comparable and scientifically sound study results. Causal inference was performed using a two-sample Mendelian randomization (MR) study, where trace elements (copper, calcium, carotene, folic acid, iron, magnesium, zinc, potassium, selenium) and vitamins (vitamin A, vitamin B12, vitamin B6, vitamin C, vitamin D, vitamin E) were considered as exposure factors, and 21 autoimmune diseases were treated as outcome factors. Single nucleotide polymorphisms (SNPs) related to micronutrients were used as instrumental variables, which satisfied the following conditions: (i) the instrumental variables are strongly associated with the exposure; (ii) the instrumental variables are not related to any confounding factors that could influence the relationship between exposure and outcome; (iii) the instrumental variables affect the outcome solely through the exposure.

### 2.3 Selection of instrumental variables

Following the STROBE-MR guidelines (21), the SNPs for each trace element and nutrient were selected through the following steps:(i)SNPs were screened using a genome-wide significance threshold of P < 5×10^-8. If the number of significant SNPs was insufficient at this threshold, a less stringent threshold of P < 5×10^-6 was used,(2)the clump function was applied to perform linkage disequilibrium (LD) testing, with set criteria of r^2 < 0.001 and a distance threshold of 10,000 kb,(3)the F-statistic for each SNP was calculated, and SNPs with F < 10 were excluded to avoid bias from weak instrumental variables. Additionally, the proportion of variance explained (R^2) by the genetic instruments was calculated to quantify the strength of the genetic instruments, using the formula:R2=[2×Beta2×(1-EAF)×EAF]/[2×Beta2×(1-EAF)×EAF+2×SE2×N×(1-EAF)×EAF],where β represents the genetic effect of each SNP, EAF is the effect allele frequency, SE is the standard error, and N is the sample size. The F-statistic for each SNP was then calculated using the formula:F=R2(N-k-1)/k(1-R2),where R2 is the proportion of exposure variance explained by the selected SNPs, N is the sample size, and k represents the number of included instrumental variables. SNPs with F < 10 were removed. The remaining independent instrumental variables were used for subsequent MR analyses.(4)The MR-PRESSO method was employed to detect outliers and adjust for horizontal pleiotropy. Any outliers identified due to horizontal pleiotropy were removed from the instrumental variables.By following these rigorous steps, the study aims to ensure the robustness and reliability of the MR analyses, thereby providing a clearer understanding of the causal effects of micronutrients on autoimmune diseases.

### 2.4 Mendelian randomization analysis

The causal relationships between micronutrients and the 21 autoimmune diseases were analyzed using the inverse variance weighted (IVW) method, the weighted median (WME) method, and MR-Egger regression. The IVW method assumes no horizontal pleiotropy among the SNPs and combines the Wald ratio estimates of each SNP to calculate the overall causal effect. The WME method, which ensures that fewer than 50% of the SNPs are invalid, reduces the occurrence of Type I errors and allows for some invalid genetic variations. MR-Egger regression incorporates the intercept term into the weighted linear regression to estimate the causal effect (slope) and the effect of horizontal pleiotropy (intercept). When instrumental variables exhibit horizontal pleiotropy, MR-Egger regression remains unaffected. The IVW method was primarily used to evaluate causal effects, with MR-Egger regression and WME methods supplementing the IVW results (24).

Sensitivity analyses included heterogeneity tests, genetic pleiotropy tests, and leave-one-out analysis. Heterogeneity was assessed using Cochran’s Q test; a P-value > 0.05 indicated no heterogeneity, whereas a P-value < 0.05 suggested potential inter-SNP heterogeneity. The ideal outcome for leave-one-out analysis was minimal change in results upon sequential removal of each SNP. Horizontal pleiotropy was evaluated using the MR-Egger intercept and MR-PRESSO global test (25). These statistical analyses were primarily conducted using the TwoSampleMR package (version 0.5.5) in R software (version 4.0.2). All analyses were performed using the R packages “TwoSampleMR” (version 0.5.6) and “MR-PRESSO” (version 1.0) in R software (version 4.3.1). The results were considered stable and meaningful if no pleiotropy and heterogeneity were detected, and the IVW, as well as other methods, yielded significant results.

Finally, this study combined multivariable Mendelian randomization (MVMR) to analyze the causal relationships between multiple exposure factors and diseases, assessing the joint effects of multiple exposures on disease risk and their interactions. Through these analyses, the study aimed to gain a deeper understanding of the causal relationships between micronutrients and the 21 autoimmune diseases, providing a scientific basis for the prevention and treatment of these diseases.

## 3 Results

### 3.1 Results of instrumental variable screening

SNPs meeting the three hypotheses were screened according to the set criteria, and the F values of the remaining instrumental variables were all >10. When the genome-wide significance threshold of P<5×10^-8^ was used as the criterion, the number of available SNPs was too small for analyzing the results, so the P value was set to P<5×10^-6^ according to the STROBE-MR study specification and the review of the literature, and 188 SNPs significantly correlated with all the micronutrients were identified as the instrumental variables. SNPs significantly associated with all trace elements were finally identified as instrumental variables.

### 3.2 Mendelian randomization results

Using the IVW method as the primary analysis, we found that elevated levels of copper (OR = 1.184, 95% CI = 1.000-1.401, P = 0.049) were associated with an increased risk of systemic lupus erythematosus, whereas higher copper levels were inversely associated with the risk of ulcerative colitis (OR = 0.939, 95% CI = 0.885-0.997, P = 0.040). Carotene levels (OR = 0.040, 95% CI = 0.003-0.480, P = 0.011) were found to have a negative causal relationship with adult-onset Still’s disease. Additionally, elevated levels of copper (OR = 1.102, 95% CI = 1.004-1.029, P = 0.041) and selenium (OR = 1.199, 95% CI = 1.036-1.389, P = 0.015) were associated with an increased risk of autoimmune hyperthyroidism. Calcium levels (OR = 0.093, 95% CI = 0.015-0.584, P = 0.011) had a negative causal relationship with the risk of polyarteritis nodosa. To avoid overestimation and ensure the reliability of the MR analysis, a series of sensitivity analyses were performed to test the robustness of the results and detect potential horizontal pleiotropy. The MR-Egger intercept indicated no evidence of horizontal pleiotropy for all causal effects (P > 0.05). Cochran’s Q test and leave-one-out analysis revealed no significant heterogeneity, indicating that the MR analysis results were robust.

Moreover, the MR study suggested that vitamin A levels were inversely associated with polymyositis (OR < 0.001, P = 0.025) and rheumatoid arthritis (OR < 0.001, P = 0.004). Elevated vitamin D levels (OR = 88.493, P = 0.005) were associated with an increased risk of interstitial lung disease related to systemic autoimmune diseases. However, we observed that the 95% confidence intervals for these three results were extremely wide, with the lower limit of the 95% CI approaching 0 and the upper limit exceeding 1000, indicating that these results may not be reliable.

### 3.3 Multivariable Mendelian randomization results

We utilized multivariable Mendelian randomization (MVMR) to analyze the causal relationships between multiple exposure factors and diseases, assessing the combined effects of multiple exposures on disease risk and their interactions. In the study on autoimmune hyperthyroidism, we evaluated the joint impact of trace elements copper and selenium as exposure factors, examining their collective influence on disease risk and their interactions. The MVMR results indicated that selenium retained a direct impact on autoimmune hyperthyroidism (OR = 1.213; 95% CI = 1.048-1.404; P = 0.010) even in the presence of copper. Sensitivity analyses revealed no evidence of horizontal pleiotropy or heterogeneity, confirming the robustness of the MR results. This suggests that selenium can independently affect the risk of autoimmune hyperthyroidism, separate from copper.

Similarly, in evaluating the causal relationships between vitamin A and both polymyositis and rheumatoid arthritis, as well as between vitamin D and interstitial lung disease related to systemic autoimmune diseases, we included all other vitamins studied as exposure factors. The results demonstrated that vitamin A could independently exert a direct influence on both polymyositis and rheumatoid arthritis. Additionally, vitamin D could independently affect the risk of interstitial lung disease associated with systemic autoimmune diseases. However, the 95% confidence intervals for the odds ratios (ORs) exhibited extreme values, leading us to consider these results as potentially unreliable.

## 4 Discussion

To the best of our knowledge, this study is the first of its kind to assess the effects of micronutrients and vitamins on autoimmune diseases through a Mendelian randomization (MR) approach. The study utilized published GWAS data to infer causal relationships between serum trace elements and vitamins and 21 autoimmune diseases. The results were analyzed to identify a number of potential causal relationships, providing new insights into the role of trace elements in autoimmune diseases.

Elevated copper (Cu) levels were found to be associated with an increased risk of systemic lupus erythematosus (SLE), which is consistent with existing literature. Patients with SLE have higher serum concentrations of Cu and serum Cu is positively correlated with C-reactive protein (CRP) (26, 27). C-reactive protein (CRP) is an acute-phase reactive protein, and its levels are usually elevated in the presence of inflammation, infection, or tissue damage in the body. CRP levels are often used as an indicator of inflammatory activity. Therefore, elevated serum copper concentrations may be associated with the inflammatory state of SLE. In addition, elevated copper levels can exacerbate the inflammatory response, and copper is known to interact with pro-inflammatory cytokines and oxidative stress pathways to increase chronic inflammation in SLE patients (28). In summary, the mechanisms by which copper affects SLE involve its role in oxidative stress and inflammation, both of which are key to the progression of the disease.

There are fewer studies on the relationship between copper intake and ulcerative colitis (UC), but in general, most of them did not find significant differences in serum copper levels between UC patients and healthy individuals. For example, none of the studies by Hussien and Zhang found an association between serum copper levels and disease in patients with UC, which may be due to the fact that elevated levels of copper-cyanin during the inflammatory response masked changes in free copper levels (29–31). Nevertheless, the independent role of copper intake in the development or severity of UC may be limited, but it has potential implications for disease management as part of the overall nutritional balance. Therefore, the overall balance of micronutrients, including copper, should be considered in the dietary management of UC. More large-scale and multicenter studies are needed in the future to further explore the relationship between copper intake and UC.

There is no clear evidence of a causal relationship between carotenoids and adult onset Still’s disease (AOSD). Although the role of carotenoids in a variety of autoimmune diseases has been explored in the literature (32, 33), there is still a lack of research support regarding their relationship with AOSD. We found that carotenoids may be negatively associated with the risk of AOSD by MR analysis, suggesting that carotenoids may play a role in the pathogenesis of AOSD, especially through the mechanisms of attenuating oxidative stress and modulating immune responses. However, further studies are needed to verify the mechanisms regarding this.

The present study suggests that selenium may be a risk factor for autoimmune hyperthyroidism (hyperthyroidism), which is in line with some of the existing findings. Selenium plays an important role in thyroid function, and studies have found that selenium deficiency may increase the risk of hyperthyroidism (34). However, our MR analysis contradicts some clinical findings. For example, selenium supplementation may improve symptoms and help delay progression in patients with Graves’ disease (35). The effect of selenium may be closely related to an individual’s baseline selenium status, dose, and form of supplementation (e.g., selenium yeast versus inorganic selenium) (36, 37). These differences suggest that the mechanism of action of selenium may be influenced by multiple factors, and more high-quality randomized controlled trials and Meta-analyses are needed in the future to further explore the relationship between selenium and hyperthyroidism.

In addition, to our knowledge, there is no clear evidence that elevated copper levels cause or are directly associated with Graves’ disease. Little is known about the relationship between copper and thyroid autoimmune diseases. It has been reported that high serum copper concentrations are positively correlated with the presence of thyroid autoantibodies (38). However, another study showed no association between copper levels and thyroid autoimmune inflammation and thyroid autoantibodies (39). One study showed no correlation between Cu concentrations and indicators of oxidative stress as well as serum levels of anti-thyroid peroxidase (anti-TPO) and anti-thyroglobulin (anti-TG) (40). Although a number of studies on the relationship between copper levels and thyroid autoimmune diseases exist, the results of these studies are inconsistent and there is no conclusive evidence to support the idea that elevated copper levels directly cause Graves’ disease. Therefore, further studies are still needed to elucidate the exact link between copper and Graves’ disease.

Regarding the relationship between calcium and tuberous arteritis (TA), our study found that calcium levels were negatively associated with the risk of TA. However, existing scientific studies are somewhat controversial regarding the relationship between calcium and arterial disease. Some studies suggest that calcium may increase the risk of cardiovascular disease through mechanisms that promote vascular calcification and affect the cardiovascular system (41–43). However, in studies of nodular arteritis, there is uncertainty regarding the role of calcium. A systematic review found inconsistencies in the correlation between serum calcium and vascular disease. Some studies showed a significant positive correlation between serum calcium and death, while others showed a significant positive correlation between serum calcium and cardiovascular disease (44). In summary, although some studies exist that show an association between calcium levels and risk of arterial disease, the current scientific findings are inconsistent and controversial. Therefore, further studies are still needed to clarify the exact relationship between calcium and arterial disease risk.

Carotenoids, as a potent antioxidant, may play a role in the pathology of adult-onset Still’s disease (AOSD) by reducing oxidative stress. Oxidative stress is considered to be a key causative factor in several autoimmune diseases, and carotenoids are capable of scavenging free radicals, thereby reducing oxidative damage. In addition, carotenoids may also play a role by modulating the immune system. Studies have shown that carotenoids have an effect on T-cell function and may regulate immune responses by promoting or inhibiting the activation of specific T-cell subsets. These mechanisms may help explain the potential relationship between carotenoids and adult-onset Still’s disease. However, the specific mechanisms regarding the role of carotenoids in this disease still need to be verified by further studies. Meanwhile, “Regarding the association between vitamin D and interstitial lung disease (ILD), we observed wider confidence intervals, which may be related to the insufficient sample size or the presence of large variability in the data. Larger variability may make the uncertainty of the results larger, leading to wider confidence intervals for the estimates. This uncertainty suggests that the result may not be statistically significant or reliable enough, so we need to interpret this finding with caution. Future studies could validate this association by expanding the sample size or using more refined analytical methods.”

Although this study provides new insights into the relationship between micronutrients and autoimmune diseases, there are some limitations to the study: First, the study was limited to a European population, and the findings may not be applicable to other populations with different genetic backgrounds, which may vary significantly across multiple different ethnic backgrounds. Second, this study is only a basic theoretical study, and more animal experiments and cohort studies are needed to confirm the conclusions for better clinical application. In addition, our findings are not entirely consistent with previous studies, and more in-depth studies are needed in the future to explore the effects of micronutrients on autoimmune diseases.

## 5 Conclusion

In conclusion, our study elucidated the causal relationship between micronutrients and nutrients and 21 autoimmune diseases through Mendelian randomization analysis. This insight provides new perspectives for subsequent mechanistic studies and new scientific basis and strategies for future clinical applications of micronutrients in the prevention and treatment of autoimmune diseases.

## Data Availability

The raw datasets generated and analysed during the current study are available in the MRCIEU GWAS database (https://gwas.mrcieu.ac.uk/) and the FinnGen R10 repository. Specific IDs were available in the data collection section of the article.
